# ΔNp63 promotes IGF1 signalling through IRS1 in squamous cell carcinoma

**DOI:** 10.18632/aging.101725

**Published:** 2018-12-28

**Authors:** Valentina Frezza, Claudia Fierro, Elena Gatti, Angelo Peschiaroli, Anna Maria Lena, Margherita Annicchiarico Petruzzelli, Eleonora Candi, Lucia Anemona, Alessandro Mauriello, Pier Giuseppe Pelicci, Gerry Melino, Francesca Bernassola

**Affiliations:** 1 Department of Experimental Medicine, TOR, University of Rome "Tor Vergata", Rome 00133 Italy; 2 Department of Experimental Oncology, European Institute of Oncology, Milan 20139 Italy; 3 National Research Council of Italy, Institute of Translational Pharmacology (IFT-CNR), Rome 00133 Italy; 4 Istituto Dermopatico dell'Immacolata, IRCCS, Rome 00163 Italy; 5 Medical Research Council, Toxicology Unit, University of Cambridge, Leicester LE1 9HN, UK

**Keywords:** p53 family, p63, IGF system, IRS1, head and neck tumours

## Abstract

Accumulating evidence has proved that deregulation of ΔNp63 expression plays an oncogenic role in head and neck squamous cell carcinomas (HNSCCs). Besides p63, the type 1-insulin-like growth factor (IGF) signalling pathway has been implicated in HNSCC development and progression. Most insulin/IGF1 signalling converges intracellularly onto the protein adaptor insulin receptor substrate-1 (IRS-1) that transmits signals from the receptor to downstream effectors, including the PI3K/AKT and the MAPK kinase pathways, which, ultimately, promote proliferation, invasion, and cell survival. Here we report that p63 directly controls IRS1 transcription and cellular abundance and fosters the PI3K/AKT and MAPK downstream signalling pathways. Inactivation of ΔNp63 expression indeed reduces tumour cell responsiveness to IGF1 stimulation, and inhibits the growth potential of HNSCC cells. In addition, a positive correlation was observed between p63 and IRS1 expression in human HNSCC tissue arrays and in publicly available gene expression data. Our findings indicate that aberrant expression of ΔNp63 in HNSSC may act as an oncogenic stimulus by altering the IGF signalling pathway.

## Introduction

Head and neck squamous cell carcinoma (HNSCC), the sixth most frequent malignancy worldwide, is a heterogeneous disease that develops from the stratified epithelium of the upper aerodigestive tract [1]. Despite recent diagnostic and therapeutic advances, the prognosis and survival of HNSCC patients remain poor. In response to the increase in the number of new HNSCC cases worldwide, further knowledge of tumour biology and the identification of novel clinical biomarkers are needed to improve prognostic stratification and optimise the anti-cancer therapies.

The p53 family of transcription factors includes p53, p63 and p73, which are all involved in tumorigenesis [2–8] as well as in fertility [9,10], metabolism [11], and aging [12–17] regulation. In addition, a developmental or differentiation function has been described for all the family members [18–32]. Many of these features are compatible with the genetic [33–37] and metabolic [38–45] events described in aging [46–55]. A common feature of the p53 family members is the presence of two distinct promoters that can be differentially used to transcribe the full-length (TA isoforms) or the N-terminal-shorter (ΔN isoforms) proteins, exerting distinct functions [56]. ΔNp63 is the predominant isoform transcribed from the TP63 gene. It is expressed in the basal compartments of several ectoderm-derived tissues [57–60], in which, it acts as a master regulator of epithelial development and maintenance [61,62]. In addition, ΔNp63 promotes the survival and sustains the self-renewal potential of epithelial cancer stem cells [63,64]. Genomic amplification and overexpression of TP63, as a result of decreased methylation of CpGs at the ΔNp63 promoter, is frequent in HNSCCs [65–67], in which ΔNp63 promotes cancer cell survival, proliferation and chemoresistance [59,66,68,69]. ΔNp63 overexpression is an adverse prognostic factor for HNSCC patients [66,70]. Although some ΔNp63 targets and molecular pathways relevant for its pro-tumorigenic activities have been identified [59,66,71,72], there still need to uncover the molecular basis of its oncogenic function.

The Insulin-like Growth Factor (IGF) axis is composed of the tyrosine kinase IGF type 1 receptor (IGF1R), its ligands, insulin, IGF1 and IGF2, the adaptor protein Insulin Receptor Substrate 1 (IRS1) and a family of six ligand-binding proteins (IGFBPs) that regulate the bioavailability and half-life of circulating IGF1. Among the IGFBPs, IGFBP3 binds to more than 95% of circulating IGF. The IGF1R binds IGF1 and IGF2 with high affinity, as well as insulin, though with lower affinity. Upon ligation, IGF1R recruits and phosphorylates IRS1. Phosphorylated IRS1 acts as docking site for intracellular adaptor proteins that, ultimately, activate two downstream signalling cascades: the PI3K/AKT and the MAPK pathways, both of which have mitogenic and pro-survival roles.

Reduced responsiveness to IGF1/insulin can occur as a result of diminished total or phosphorylated amounts of IGF1R or IRS1, and results in decreased activation of the PI3K/AKT and MAPK pathways [73].

Deregulation of the IGF axis has been implicated in the development and progression of several human cancers. Elevated serum IGF1 levels, and increased levels or constitutive activation of IGF1R and IRS1 are associated with increased risk of a variety of epithelial cancers, metastasis and therapeutic resistance [74–80]. Hence, it has been proposed that reduction of IGF signalling may have a prognostic impact and a therapeutic benefit in some cancer types. In HNSCC, IGF1R overexpression is associated with adverse survival, HPV negativity and high tumour T-stage [76,81,82]. High IGF1 levels and both lower and higher levels of IGFBP3 are predictor risk factors for secondary tumour development in patients with HNSCC [83,84]. In addition, increased IRS1 expression was found in nasopharyngeal carcinoma patients, where it correlated with lymph node metastasis [80].

Notably, the existence of a possible crosstalk between p63 and the IGF system has been reported. The tumour suppressive TAp63 isoforms negatively control *Igf1r* transcription [85], whereas *Igfbp3* is a target of transcriptional repression by ΔNp63 [86]. Here we report, that Np63 affects the transcription and the cellular abundance of *Irs1* in HNSCC cells and, that, as a result, p63 down-regulation impairs the activation of the intracellular signalling pathways following IGF1R stimulation.

## RESULTS

### p63 controls IRS1 expression levels in HNSCC cells

**Figure 1:**
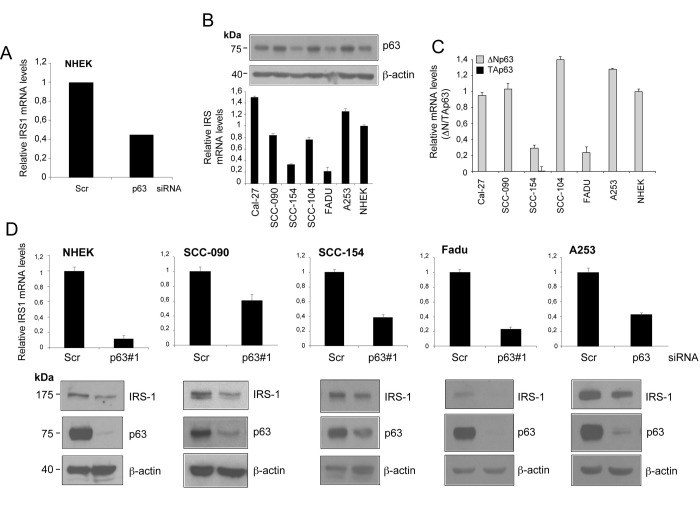
**IRS1 expression is decreased upon down-regulation of p63 in HNSCC cell lines.** (**A**) Relative expression levels of *Irs1* as measured by RNA-Seq analysis of p63-depleted NHEK. Cells were transfected with p63 (sip63#1) or scrambled control (siScr) siRNAs. P-value = 0,005. (**B**) The amount of p63 was measured in NHEK and HNSCC cell lines by western blot analysis (upper panel). IRS1 transcript levels were analysed by RT-qPCR (lower panel). RT-qPCR was performed in duplicate. IRS1 expression was normalized on *Tbp* housekeeper and plotted relative to NHEK cells (mean ± s.d.). (**C**) The transcript levels of TAp63 (black box) and ΔNp63 (grey box) were measured in NHEK and HNSCC cell lines by RT-qPCR. RT-qPCR was performed as above. Gene expression was normalized on *Tbp* housekeeper and plotted relative to NHEK cells (mean ± s.d.). (**D**) RT-qPCR analysis (upper panels) of two independent experiments performed in duplicates for *Irs1* transcripts in NHEK and HNSCC cells transfected with scrambled control (siScr) or p63 (sip63#1) siRNAs. Cells were harvested 48 h after transfection. qRT-PCR was performed as above. Values are normalized to *Tbp* and plotted relative to control cells (mean ± s.d.). Western blot analysis for IRS1 and p63 in HNSCC cells transfected as above. Cells were harvested 48 h after transfection. β-actin served as loading control.

By exploiting RNA sequencing (RNA-seq) transcriptome profiling, we identified genes regulated by ΔNp63 in normal human epidermal keratinocytes (NHEKs) (E.C. unpublished data). The analysis of RNA-Seq data obtained from p63-depleted cells revealed almost 50% reduction in IRS1 expression levels relatively to control cells (Fig. 1A). We then sought to test whether IRS1 expression is regulated by p63 in HNSCC cells. HNSCC cell lines display moderate/high levels of p63 expression (Fig. 1B, upper panel). In particular, the predominantly expressed isoform of p63 in HNSCC cells is ΔNp63, with TAp63 being undetectable in the majority of the cell lines (Fig. 1C). We observed a consistent correlation between ΔNp63 protein levels and the gene expression pattern of IRS1 in most of the HNSCC cell lines analysed (Fig. 1B, lower panel). Validation of RNA-seq data showed that, following p63 knockdown, IRS1 transcript and protein levels were reduced in NHEK and in a panel of HNSCC cell lines (Fig. 1D).

### p63 induces IRS1 expression by binding directly to the regulatory region of the *Irs1* gene

**Figure 2:**
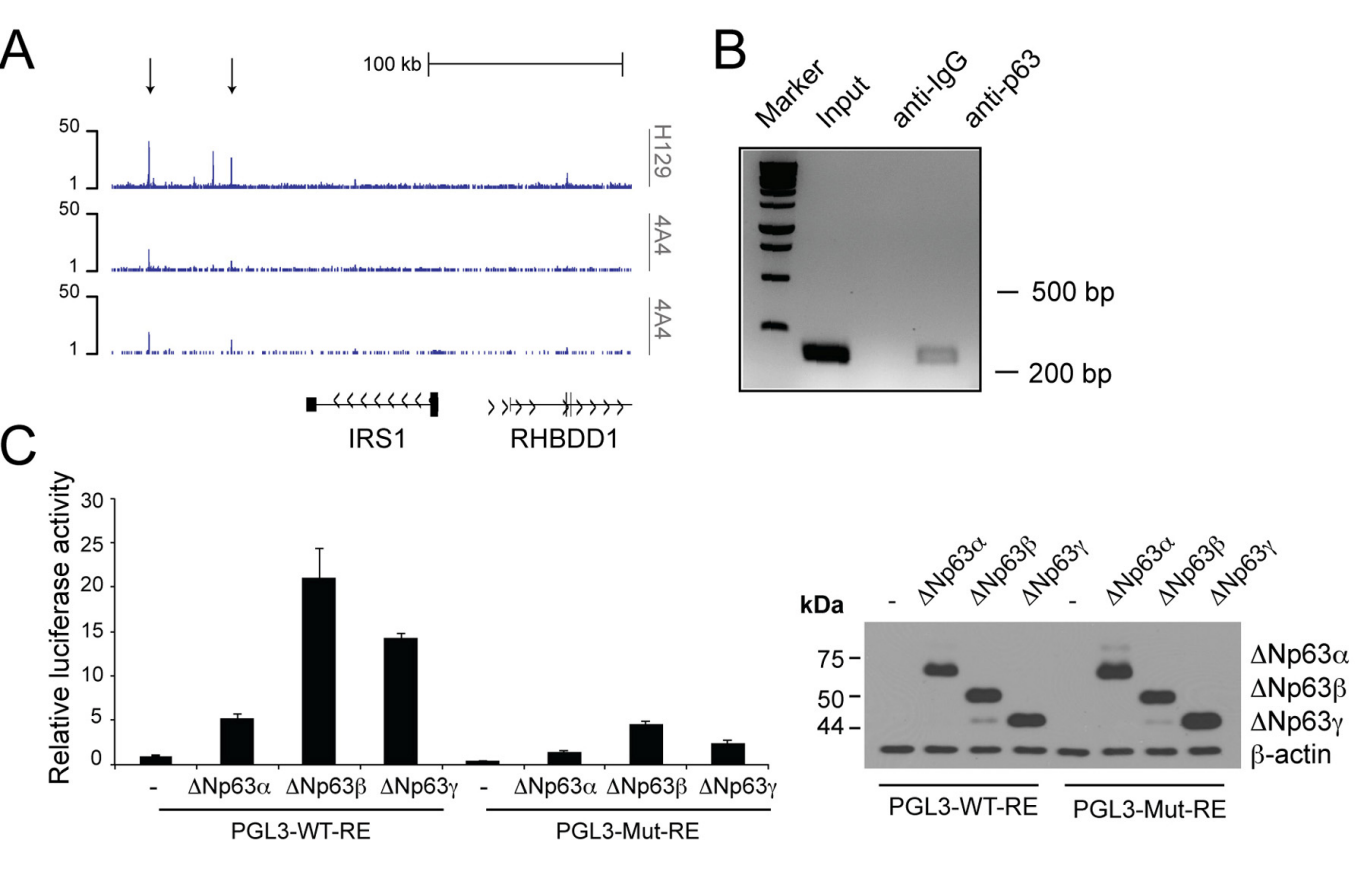
**p63 binds to the regulatory region of the** ***Irs1*** **gene.** (**A**) p63 DNA-binding profiles in the *Irs1* locus, obtained in NHEKs by ChIP-sequencing (ChIP-seq) using 4A4 and H129 anti-p63 antibodies in two normal human primary keratinocyte cell lines (K1 and K2) [87]. (**B**) ChIP analysis of p63 occupancy at the regulatory regions of the *Irs1* gene. ChIP assays were performed in Fadu HNSCC cells using H129 anti-p63 antibody and control IgGs. PCR validation was performed using primers spanning the p63-binding sites located within the genomic regions identified by ChIP-seq assays. (**C**) Luciferase reporter assays of *Irs1* regulatory regions (left panel). The pGL3 reporter vector (30 ng) and the pRL-CMV-*Renilla* luciferase plasmid (5 ng) were cotransfected with the empty pcDNA-HA vector or plasmids coding ΔNp63α, ΔNp63β, and ΔNp63γ (150 ng) into the p53 null human H1299 cell line. The luciferase activities of cellular extracts were measured 24 h after transfection. Cellular lysates were also analysed by western blot (right panel). Data are presented as mean ± SD and are representative of three independent experiments.

Genome-wide profiling of p63 binding sites by Chromatin IP Sequencing (ChIP-seq) analysis of NHEKs [87] revealed peaks of p63 binding to regions downstream the *Irs1* locus (Fig. 2A). The algorithm p63scan identified a putative p63 responsive element (RE) in the most distant enriched peak. To validate direct interaction of p63 with this putative RE, we examined p63 occupancy at the site identified in the ChIP-seq analysis. By ChIP experiments in HNSCC cells, we found binding of p63 to a regulatory region located downstream the *Irs1* locus (+148 kbps from the TSS) (Fig. 2B). In addition, by performing luciferase activity reporter assays in H1299 cells, we found that the ΔNp63 isoforms activate a luciferase reporter gene driven by the p63 RE located in the regulatory region of the *Irs1* locus (Fig. 2C). Site-specific mutagenesis of the p63 RE almost completely abrogated the transactivating ability of ΔNp63 (Fig. 2C). Overall, these data demonstrate that *Irs1* is a direct transcriptional target of ΔNp63.

### p63 inactivation impairs cellular sensitivity of HNSCC cells to IGF1/insulin stimulation

**Figure 3:**
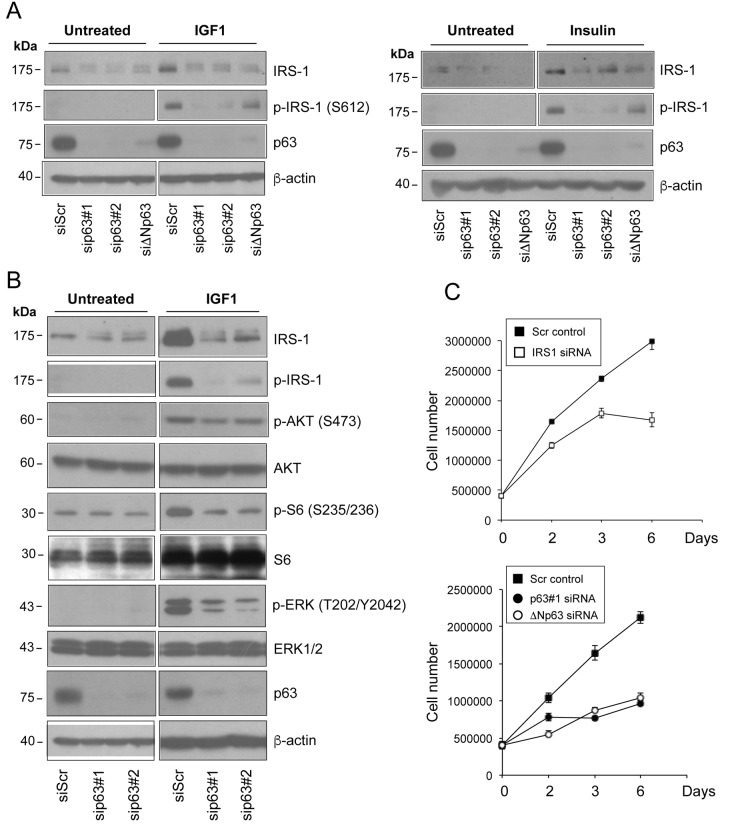
D**epletion of p63 reduces the responsiveness of HNSCC cells to ligand stimulation.** (**A**) Fadu cells were transfected with siScr or different p63 (sip63#1, sip63#2, siΔNp63) siRNAs. Forty-eight h after transfection, cells were serum starved for 4 h, and then stimulated with 5 nM IGF1 (upper panel) or 500 ng/ml insulin (lower panel) for 10 min. Protein amounts of p63, IRS1 and p-IRS1 were detected by western blot analysis. β-actin served as loading control. Blots are representative of three individual experiments. (**B**) Fadu cells were transfected with siScr, sip63#1 and sip63#2, serum starved for 4 h and then stimulated with 5 nM IGF1 for 10 min. Cellular extracts were analysed with the following antibodies: anti-IRS1, anti-p-IRS1, anti-p-AKT, anti-AKT, anti-p-S6 Ribosomal Protein, anti-S6, anti-p44/42 MAPK (p-ERK1/2), anti-ERK1/2, p63 and β-actin as loading control. Blots are representative of three individual experiments. (**C**) Fadu cells were transfected with siScr or siIRS1 (upper panel) and with sip63#1, ΔNp63, or siScr (lower panel). Forty-eight h after transfection, cells were seeded in 6-cm plates at 500,000/plate and growth was followed until day 6.

We next tested whether knockdown of ΔNp63 affects the level of activated IRS1. To assess whether down-regulation of IRS1 in p63-depletd HNSCC cells would impair cellular responsiveness to receptor stimulation, we treated serum starved Fadu cells with both IGF1 and insulin. Stimulation of the IGF1R resulted in reduced levels of phosphorylated (Ser612) IRS1 in p63-depleted relatively to control cells (Fig. 3A). To rule out off-target effects two independent siRNAs against p63 and one specific ΔNp63 siRNA were employed (Fig. 3A, Fig. S1A and S1B).

To examine whether alteration of IRS1 and phospho-IRS1 levels induced by p63 silencing may affect downstream IGF signal transduction, we measured the activation of PI3K/AKT and MAPK downstream signalling pathways in p63-depleted cells. Upon p63 knock-down, we observed desensitization of HNSCC cells to IGF1 stimulation, as assessed by decreased amounts of phospho-AKT (Ser473) and phospho-S6 (Ser235/236) (Fig. 3B). Activation of MAPK (phospho-Erk1/2; Thr202/Tyr204) signalling pathway in response to IGF-1 stimulation was also markedly reduced in p63-depleted cells (Fig. 3B), further proving that p63 affects cellular sensitivity to IGF1/insulin stimulation through the regulation of IRS1 cellular abundance. Notably, knockdown of IRS1 hampered the proliferation of HNSCC cells, mimicking the effect of p63 inactivation (Fig. 3C). These findings indicate that the p63/IRS1 functional axis positively regulates the growth potential of HNSCC cells.

### p63 and IRS1 expression correlates in HNSCC patients

**Figure 4:**
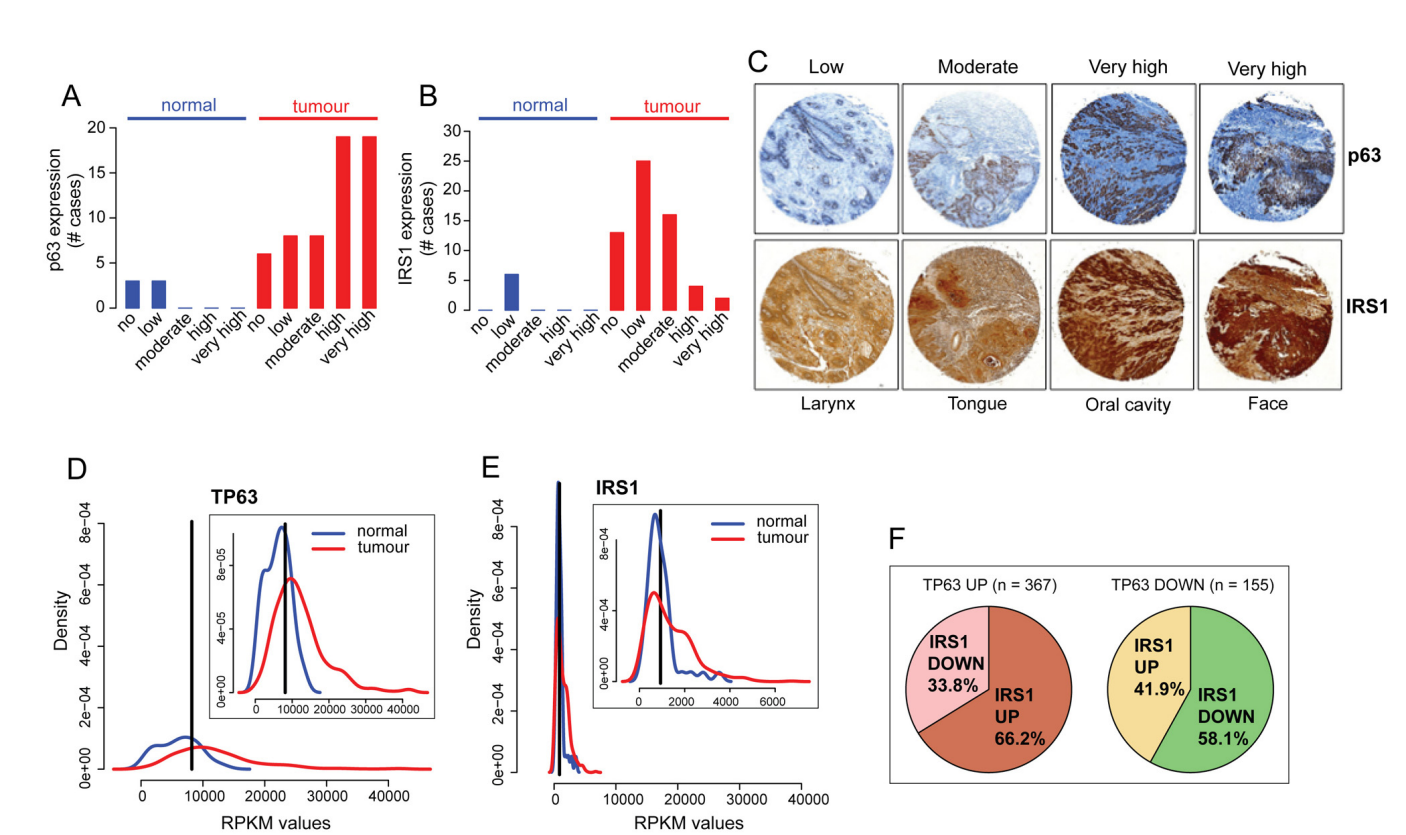
**p63 and IRS1 expression patterns are positively correlated in HNSCC patients**. (**A-C**) Tissue sections were stained with anti-p63 and IRS1 antibodies. The scores for the percentage of p63 and IRS1 positive cells and those for the expression intensities were combined to calculate immunoreactive scores (IRSs) summarised in Table S1. According to the IRS, patients were assigned to five groups: 0 (no expression), 0,5 (low expression), 1 (moderate expression), 1,5-2 (high expression), and 2,5-3 (very high expression). The distribution of p63 and IRS1 expression in HNSCC specimens and normal tissues is shown in **A, B**. (**C**) Representative micrographs of immunohistochemical staining of p63 and IRS1 in HNSCC primary tumours (magnification: 40x). (**D-E**) Density distribution of RPKM values in normal (n=44; blue) and tumour (n=522: red) samples for p63 and IRS1 transcripts. Top right panels show an enlargement of the overlapping curves. Vertical black line indicates the optimal cutpoint between tumour and normal RPKM distributions, identified using *OptimalCutpoints* R package [96] *P-value* < 1.074e-10 for p63 and *P-value* < 0.004 for IRS1. (**F**) Pie charts representing the proportion of tumour samples with IRS1 up- or down-regulated in p63 up (left) and p63 down-regulated (right) tumour samples. Statistical significance of the contingency table represented by the 4 sub-dataset of tumour samples is p-value= 4.293e-07 (Fisher’s exact test). Statistical tests (Wilcoxon and Fisher’s exact tests) on TCGA gene expression data were performed in R.

To examine possible correlations between the expression levels of ΔNp63 and IRS1 in NHSCC primary tumours, clinical NHSCC tumour specimens samples and related benign controls were examined for p63 and IRS1 staining on tissue microarray slides. Consistent with previous reports [65,66], the majority of the patients (63%) showed high levels of p63 expression (Fig. 4A; representative staining patterns are seen in Fig. 4C, top panels). High and moderate tumour cell IRS1 expression was observed in 27% and 10%, respectively of the cases (Fig. 4B, representative staining patterns are seen in Fig. 4C, bottom panels). Significant correlation of p63 and IRS1 expression was observed in 23 out of 60 HNSCC samples (38%, Fig. 4C and Table S1).

To further investigate the expression levels of p63 and IRS1, we analysed publicly available transcriptome sequencing data of 522 HNSCC patients from the TCGA repository [88]. Overall, the transcript levels of p63 and IRS1 are significantly higher in tumour specimens than in normal samples (Fig. 4D, and 4E). On the basis of their expression levels, for each gene, we stratified tumour patients into two distinct groups, displaying either up-regulation or down-regulation relatively to normal subjects. Notably, we found that 66.2% of tumour samples with p63 up-regulation also exhibited high levels of IRS1 expression A similar correlation was observed in samples with p63 down-regulation, in which 58% of the patients concomitantly displayed low levels of IRS1 transcripts (Fig. 4F, p-value= 4.293e-07). Overall, these findings demonstrate that a statistical significant positive association exists between p63 and IRS1 expression in HNSCC patients.

## DISCUSSION

It has been originally hypothesized that, ΔNp63 mainly exerts its oncogenic functions by acting as a dominant negative repressor of the tumour suppressive members of the p53 family, including TAp63. As a consequence of preventing access to their DNA binding sites, ΔNp63 would impinge on the transcription of genes involved in cell cycle control and cell death. In addition, emerging evidence has unveiled key tumour-related signalling pathways that are transcriptionally regulated by ΔNp63, in a p53 independent manner [89]. For instance, by acting in concert with the chromatin remodelling factor ACTL6A, p63 controls chromatin accessibility and functions as a direct transcriptional repressor of the Hippo/YAP regulator WWC1 in SCC [66]. Furthermore, ΔNp63 controls a transcriptional program comprising the hyaluronic acid (HA) synthase HAS3 and two hyaluronidase genes, HYAL-1 and HYAL-3, thus sustaining the pro-tumorigenic HA metabolism and signalling [59]. Abraham and collaborators [90] have recently identified components of the transforming growth factor-β signalling and the RHOA GTPase as targets and mediators of ΔNp63-dependent cell proliferation in SCCs.

**Figure 5:**
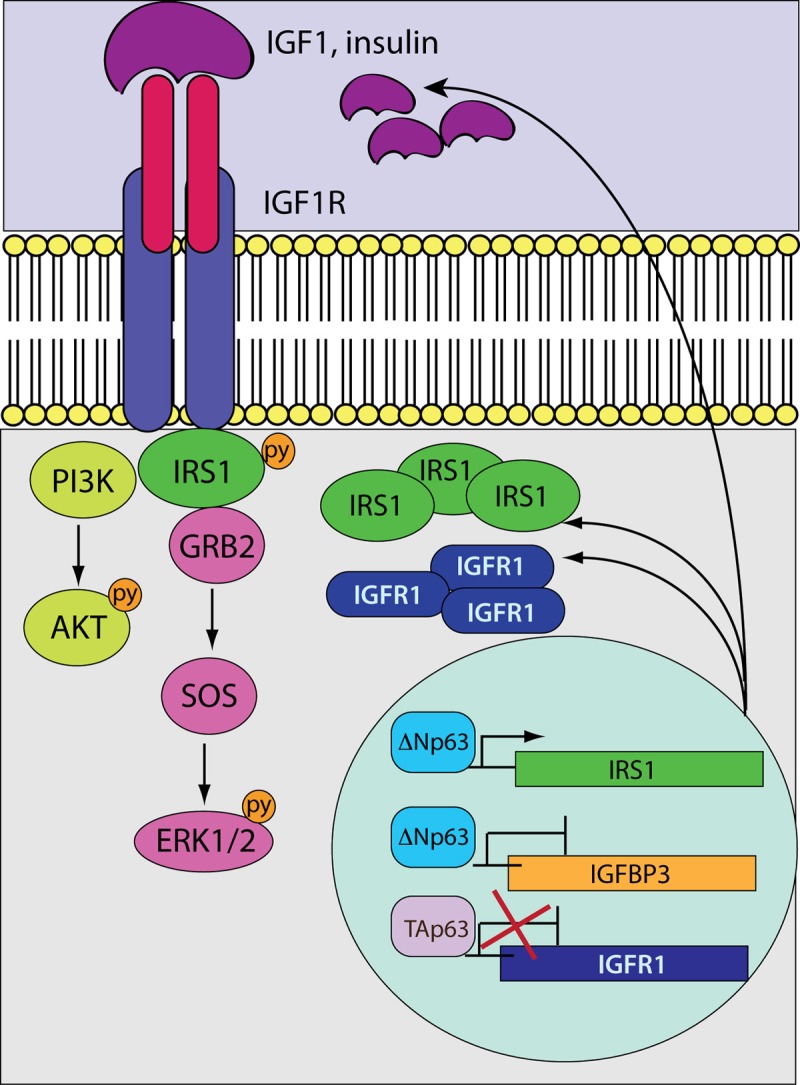
**A model depicting the crosstalk between p63 and the IGF system**. While the tumour suppressive TAp63 isoforms negatively control *Igf1r* transcription, the oncogenic ΔNp63 variants induce and repress the expression of the *Irs1* and *Igfbp3* genes, respectively. In HNSCC cells overexpressing ΔNp63, the transcription of *Igf1r* would be stimulated as a result of the unbalanced ratio between the TA/ΔN p63 proteins. Aberrant accumulation of IGF1R and its docking protein IRS1 would enhance signalling activation in response to receptor stimulation. On the other hand, reduced expression levels of *Igfbp3* would increase the availability of circulating IGF1 that could further potentiate receptor activation.

Our data demonstrate that IRS1 is a direct target of transcriptional activation by ΔNp63 in HNSCC cells. Coherently, ΔNp63 and IRS1 expression patterns are positively correlated in primary tumours, suggesting that the interaction of p63 and IRS1 might contribute to the pathogenesis of HNSCC. More broadly, in ΔNp63 overexpressing SSC tumours, unbalanced expression of the TAp63/ΔNp63 isoforms may lead to enhanced *Igf-r1/Irs1* transcriptional activation, resulting in augmented protein abundance of IGFR1 and IRS1, which may increase sensitivity of cancer cells to growth factor stimulation (Figure 5). In addition, overexpression of ΔNp63 prevents expression of *Igfbp3* thus, ultimately, enhancing circulating IGF-1 (Fig. 5). Thus, the existence of a crosstalk between p63 and the IGF1 system may represent a mechanism by which tumours that overexpress ΔNp63 escape apoptosis and acquire a proliferative advantage.

Elevated levels of IRS1 have been reported to contribute to cancer development and progression [78,80,91,92]. Notably, high expression of IRS1 in breast cancer cells was positively correlated with aberrant phosphorylation of AKT, which was significantly associated with lymph node metastasis [93]. Coherently, we observed reduced ligand-stimulated activation of AKT in p63-depleted cells, implying that reducing the cellular abundance of IRS1 could be a strategy to diminish the mitogenic potential of the IGF system. Acting as an adaptor protein that conveys signals originating from different receptors to multiple downstream signalling molecules, IRS1 represents a potentially relevant predictive clinical biomarkers for cancers susceptible to IGF-IR targeting.

Several reports have showed a correlation between the IGF pathway and HNSCC clinical parameters [80–84]. On the other hand, IGF pathway-related proteins have not been implemented as cancer biomarkers yet, due to the existence of contradictory findings on their prognostic impact. Indeed, negative correlations between levels of IGF1/IGF1R and clinical outcomes have been also reported in HNSCC [94]. Thus, in addition to unveiling alterations within the IGF system in tumours, the identification of alterations outside the IGF axis (*e.g.* upstream regulators), which may affect the IGF signalling, would be relevant to establish additional predictive markers for patient stratification and clinical management.

The controversial clinical data on the prognostic impact of the IGF system in HNSCC may reflect the high histopathological heterogeneity within this disease that includes tumours arising from various anatomical sites. Whether or not deregulation of the IGF signalling network and the positive regulation of IRS1 by ΔNp63 may have a prognostic significance for HNSCC are still relevant questions in the field, and further studies are needed to clarify these issues.

## MATERIALS AND METHODS

### Cells and culture conditions

Neonatal normal human epidermal keratinocytes (NHEKs, Life Technologies) were cultured in EpiLife medium with human keratinocyte growth supplements added (Life Technologies). FaDu (pharynx squamous cell carcinoma), SSC-090 (oral squamous cell carcinoma) and SSC-154 (tongue squamous cell carcinoma) cells were grown in Eagle’s minimum essential medium (EMEM, Lonza, Basel, Switzerland); A253 cells (submaxillary salivary gland carcinoma) were cultured in McCoy’s medium (Gibco, Invitrogen); SCC-9 cells (tongue squamous cell carcinoma) were cultured in1:1 mixture of Dulbecco's Modified Eagle's Medium (DMEM) and Ham’s F-12 medium (DMEM F12, Gibco, Invitrogen) supplemented with 400 ng/mL hydrocortisone; CAL27 (tongue squamous cell carcinoma) and SCC-104 (oral squamous cell carcinoma) cells were grown in DMEM. All media were supplemented with 10% FBS, 100 U penicillin, and 100 μg/mL streptomycin (Gibco, Life Technologies). All HNSCC cell lines were purchased from ATCC and routinely tested for mycoplasma contaminations.

For p63 siRNA-mediated knockdown, NHEK and HNSCC cells were transfected with the following siRNAs: sip63#1 (SASI_Hs02_00326864) and sip63#2 (SASI_Hs02_00326867) oligos were purchased from Sigma-Aldrich; the sense strand of the siΔNp63 is: 5’- GAAGAAAGGACAGCAGCATTG -3’. The Negative Control siRNA (Qiagen, AATTCTCCGAACGTGTCACGT) was used as a silencing control. All transfections were performed using the Lipofectamine RNAiMAX transfection reagent (Invitrogen) according to manufacturer's protocols.

### RNA sequencing

RNA sequencing was performed as previously described [95]. Briefly, total RNA was extracted using a mirVana miRNA isolation kit (Thermo Fisher). rRNA was removed from each RNA extraction before proceeding with RNA seq library construction. Sequencing was performed on a SOLiD sequencer 5500XL (Applied Biosystems) with 75-base-pair single-end reads by Genomnia s.r.l. (Milan, Italy). Sequencing reads in SOLID “xsq” format were mapped to the hg19 genome built and analysed with the Lifetech Lifescope 2.5.1 Whole Transcriptomic analysis pipeline with the Integromics Seqsolve software and proprietary Genomnia procedures.

### Real-time qPCR

Total RNA was extracted by the RNAeasy kit (Qiagen, Hilden, Germany). Total RNA (500ng) was used for reverse transcription using GoScript Reverse Transcription System kit (Promega, Fritchburg, WI, USA) following the manufacturer's instructions. Human *Tbp* mRNA was used as housekeeping gene for quantity normalization. qRT-PCR was performed using the Platinum SYBR Green qPCR SuperMix UDG (Invitrogen), using the following primer pairs: *Irs1* 5′- CTCAACTGGACATCACAGCAG -3′ (sense) and 5′- AGGTCCTAGTTGTGAATCATG -3′ (antisense); *TAp63* 5′- TCAGAAGATGGTGCGACAAAC -3′ (sense) and 5′- GTTCAGGAGCCCCAGGTTCG-3′ (antisense); *ΔNp63* 5′- GAAGAAAGGACAGCAGCATTG -3′ (sense) and 5′- GGGACTGGTGGACGAGGAG -3′ (antisense). The PCR was monitored by a melting curve protocol according to the specifications of the ABI 7500 instrument (Applied Biosystems). Relative quantification of gene expression was calculated according to the 2^−ΔΔCt^ method. IRS1 expression was normalized on *Tbp* housekeeper.

### Western blot analysis

Cells were lysed in SDS lysis buffer (100 mM Tris рН 8.8, 1% SDS, 5 mM EDTA, 20 mM DTT, and 2 mM AEBSF). Total protein extracts were resolved in SDS polyacrylamide gel and blotted onto a Hybond PVDF membrane (GE Healthcare, Chicago, IL, USA). After being blocked with PBST 5% non-fat dry milk (Bio-Rad), membranes were incubated over night with primary antibodies at +4°C, washed and hybridized for 1 h at room temperature using the appropriate horseradish peroxidase-conjugated secondary antibody (rabbit and mouse, Bio-Rad, Hercules, California, USA). Detection was performed with the ECL chemiluminescence kit (Perkin Elmer, Waltham, Massachusetts, USA). The following antibodies were used: anti-IRS1 (D32G12, Cell Signaling), anti-p63 (clone 4A4, Santa Cruz Biotechnology), anti-p-IRS1 (Ser612, clone C15H5, Cell Signaling), anti-p-AKT (Ser473, clone D9E, Cell Signaling), anti-AKT (clone #9272, Cell Signaling), anti-p-S6 Ribosomal Protein (Ser235/236, clone #2211, Cell Signaling), anti-S6 (Clone 5G10, Cell Signaling), anti-p44/42 MAPK (p-ERK1/2) (Thr202/Tyr204, Clone E10, Cell Signaling), anti-ERK1/2 (Clone 137F5, Cell Signaling).

### Chromatin immunoprecipitation assay

Fadu cells were used for ChIP assay. Cells were collected, fixed in 1% formaldehyde, and subjected to sonication for DNA shearing. The ChIP assay was performed with an anti-p63 antibody (H129, Santa Cruz Biotechnology) or unspecific immunoglobulin G (IgG) (Invitrogen) using a ChIP assay Kit (Invitrogen). PCR validation was performed using primers spanning the p63-binding sites located within the genomic regions identified by ChIP-seq assays.

### Luciferase assay

For luciferase assays, a total of 1.2×10^6^ H1299 cells were seeded in 12-well dishes 24 h before transfection. In total, 30 ng of pGL3 reporter vector, 5 ng of pRL-CMV-*Renilla* luciferase vector (Promega) and 150 ng of HA-ΔNp63α expression vectors or empty pcDNA-HA vector (as a control) were cotransfected using the Effectene transfection reagent according to the manufacturer's instructions (Qiagen). The luciferase activities of cellular extracts were measured 24 h after transfection using a Dual Luciferase Reporter Assay System (Promega). The light emission was measured over 10 sec using a Lumat LB9507 luminometer (EG&GBerthold). The transfection efficiency was normalized to *Renilla* luciferase activity. The p63 RE was mutated by site-directed mutagenesis using the forward primer: 5’-ATAAGGGCCTTCCTGTTCCGAGGCAGCCGTGTGAACCACC-3’ and reverse primer: 5’- GGTGGTTCACACGGCTGCCTCGGAACAGGAAGGCCCTTAT-3’.

### Human HNSCC tumour tissues

Tissue arrays including paraffin-embedded HNSCC (n=60), adenoid cystic carcinomas (n=7), adenocarcinomas (n=1), mucoepidermoid carcinomas (n=1), and normal tissues. (n=10) were purchased from US Biomax, Inc. (Rockville, MD). Samples were deparaffinized and rehydrated in 1x PBS for 10 min. Tissue sections were stained with anti-p63 (clone 4A4, Ventana) and IRS1 (Clone EP263Y, Abcam) antibodies. Immunoistochemistry (IHC) scoring was performed by two independent pathologists. The percentage of positive cells was rated as follows: 0, negative; 1, 1–25% positive cells; 2, 26–50% positive cells; 3, 51–75% positive cells; and 4, >75% positive cells. The staining intensity was scored as 0, negative; 1, weak; 2, moderate; and 3, intensive. For both stainings, the scores for the percentage of positive cells and those for the expression intensities were combined to calculate immunoreactive scores (IRSs) that are summarised in Table S1. According to the IRS, we grouped patients into five groups: 0 (no expression), 0,5 (low expression), 1 (moderate expression), 1,5-2 (high expression), and 2,5-3 (very high expression). Non squamous cell carcinomas of the head and neck were excluded from the analysis.

### Computational methods

Publicly available gene expression data from TCGA were downloaded from the Genomic Data Commons (GDC) Data Portal and pre-processed via the *TCGAbiolinks* R package [96]. Harmonized and normalized RPKM data from GDC was downloaded for Head and Neck Squamous Cell Carcinomas (TCGA-HNSC).

RPKM distributions for each of the genes of interest have been analyzed either for tumor and normal samples in the HNSC cohort. A discrimination cutpoint between tumor and normal expression distributions either for TP63 and IRS1 has been detected using *OptimalCutpoints* R package [97]. The identified optimal cutoffs represent the RPKM values that maximize the separation of gene expression distributions in tumor and normal samples. Data mining, statistical tests (Wilcoxon and Fisher’s exact tests) and plots on TCGA gene expression data were performed in R.

### Statistical analysis

The significance of differences between two experimental groups was calculated using the two-tailed Student’s t-test. Values with *P*<0.05 were considered significant.

## Supplementary Material

Supplementary File

## References

[1] Lin MC, Chien PH, Wu HY, Chen ST, Juan HF, Lou PJ, Huang MC. C1GALT1 predicts poor prognosis and is a potential therapeutic target in head and neck cancer. Oncogene. 2018; 37:5780–93. 10.1038/s41388-018-0375-0 29930379 PMC6202324

[2] Aggarwal M, Saxena R, Sinclair E, Fu Y, Jacobs A, Dyba M, Wang X, Cruz I, Berry D, Kallakury B, Mueller SC, Agostino SD, Blandino G, et al. Reactivation of mutant p53 by a dietary-related compound phenethyl isothiocyanate inhibits tumor growth. Cell Death Differ. 2016; 23:1615–27. 10.1038/cdd.2016.48 27258787 PMC5041190

[3] Amelio I, Knight RA, Lisitsa A, Melino G, Antonov AV. p53MutaGene: an online tool to estimate the effect of p53 mutational status on gene regulation in cancer. Cell Death Dis. 2016; 7:e2148. 10.1038/cddis.2016.42 26986515 PMC4823943

[4] Alexandrova EM, Moll UM. Depleting stabilized GOF mutant p53 proteins by inhibiting molecular folding chaperones: a new promise in cancer therapy. Cell Death Differ. 2017; 24:3–5. 10.1038/cdd.2016.145 27935583 PMC5260503

[5] Ghosh S, Salot S, Sengupta S, Navalkar A, Ghosh D, Jacob R, Das S, Kumar R, Jha NN, Sahay S, Mehra S, Mohite GM, Ghosh SK, et al. p53 amyloid formation leading to its loss of function: implications in cancer pathogenesis. Cell Death Differ. 2017; 24:1784–98. 10.1038/cdd.2017.105 28644435 PMC5596421

[6] Flores ER, Sengupta S, Miller JB, Newman JJ, Bronson R, Crowley D, Yang A, McKeon F, Jacks T. Tumor predisposition in mice mutant for p63 and p73: evidence for broader tumor suppressor functions for the p53 family. Cancer Cell. 2005; 7:363–73. 10.1016/j.ccr.2005.02.019 15837625

[7] Galluzzi L, Vitale I, Aaronson SA, Abrams JM, Adam D, Agostinis P, Alnemri ES, Altucci L, Amelio I, Andrews DW, Annicchiarico-Petruzzelli M, Antonov AV, Arama E, et al. Molecular mechanisms of cell death: recommendations of the Nomenclature Committee on Cell Death 2018. Cell Death Differ. 2018; 25:486–541. 10.1038/s41418-017-0012-4 29362479 PMC5864239

[8] Gressner O, Schilling T, Lorenz K, Schulze Schleithoff E, Koch A, Schulze-Bergkamen H, Lena AM, Candi E, Terrinoni A, Catani MV, Oren M, Melino G, Krammer PH, et al. TAp63alpha induces apoptosis by activating signaling via death receptors and mitochondria. EMBO J. 2005; 24:2458–71. 10.1038/sj.emboj.7600708 15944736 PMC1173149

[9] Inoue S, Tomasini R, Rufini A, Elia AJ, Agostini M, Amelio I, Cescon D, Dinsdale D, Zhou L, Harris IS, Lac S, Silvester J, Li WY, et al. TAp73 is required for spermatogenesis and the maintenance of male fertility. Proc Natl Acad Sci USA. 2014; 111:1843–48. 10.1073/pnas.1323416111 24449892 PMC3918781

[10] Levine AJ, Tomasini R, McKeon FD, Mak TW, Melino G. The p53 family: guardians of maternal reproduction. Nat Rev Mol Cell Biol. 2011; 12:259–65. 10.1038/nrm3086 21427767

[11] Marini A, Rotblat B, Sbarrato T, Niklison-Chirou MV, Knight JR, Dudek K, Jones C, Bushell M, Knight RA, Amelio I, Willis AE, Melino G. TAp73 contributes to the oxidative stress response by regulating protein synthesis. Proc Natl Acad Sci USA. 2018; 115:6219–24. 10.1073/pnas.1718531115 29844156 PMC6004440

[12] Baldelli S, Ciriolo MR. Altered S-nitrosylation of p53 is responsible for impaired antioxidant response in skeletal muscle during aging. Aging (Albany NY). 2016; 8:3450–67. 10.18632/aging.101139 28025407 PMC5270679

[13] Bourgeois B, Madl T. Regulation of cellular senescence via the FOXO4-p53 axis. FEBS Lett. 2018; 592:2083–97. 10.1002/1873-3468.13057 29683489 PMC6033032

[14] Guo X, Keyes WM, Papazoglu C, Zuber J, Li W, Lowe SW, Vogel H, Mills AA. TAp63 induces senescence and suppresses tumorigenesis in vivo. Nat Cell Biol. 2009; 11:1451–57. 10.1038/ncb1988 19898465 PMC2920298

[15] Regina C, Compagnone M, Peschiaroli A, Lena AM, Melino G, Candi E. ΔNp63α modulates histone methyl transferase SETDB1 to transcriptionally repress target genes in cancers. Cell Death Discov. 2016; 2:16015. 10.1038/cddiscovery.2016.15 27551509 PMC4979509

[16] Rivetti di Val Cervo P, Lena AM, Nicoloso M, Rossi S, Mancini M, Zhou H, Saintigny G, Dellambra E, Odorisio T, Mahé C, Calin GA, Candi E, Melino G. p63-microRNA feedback in keratinocyte senescence. Proc Natl Acad Sci USA. 2012; 109:1133–38. 10.1073/pnas.1112257109 22228303 PMC3268329

[17] Rufini A, Niklison-Chirou MV, Inoue S, Tomasini R, Harris IS, Marino A, Federici M, Dinsdale D, Knight RA, Melino G, Mak TW. TAp73 depletion accelerates aging through metabolic dysregulation. Genes Dev. 2012; 26:2009–14. 10.1101/gad.197640.112 22987635 PMC3444727

[18] Artigas N, Gámez B, Cubillos-Rojas M, Sánchez-de Diego C, Valer JA, Pons G, Rosa JL, Ventura F. p53 inhibits SP7/Osterix activity in the transcriptional program of osteoblast differentiation. Cell Death Differ. 2017; 24:2022–31. 10.1038/cdd.2017.113 28777372 PMC5686339

[19] Belle JI, Petrov JC, Langlais D, Robert F, Cencic R, Shen S, Pelletier J, Gros P, Nijnik A. Repression of p53-target gene Bbc3/PUMA by MYSM1 is essential for the survival of hematopoietic multipotent progenitors and contributes to stem cell maintenance. Cell Death Differ. 2016; 23:759–75. 10.1038/cdd.2015.140 26768662 PMC4832099

[20] Agostini M, Romeo F, Inoue S, Niklison-Chirou MV, Elia AJ, Dinsdale D, Morone N, Knight RA, Mak TW, Melino G. Metabolic reprogramming during neuronal differentiation. Cell Death Differ. 2016; 23:1502–14. 10.1038/cdd.2016.36 27058317 PMC5072427

[21] Charni M, Aloni-Grinstein R, Molchadsky A, Rotter V. p53 on the crossroad between regeneration and cancer. Cell Death Differ. 2017; 24:8–14. 10.1038/cdd.2016.117 27768121 PMC5260496

[22] Di Franco S, Sala G, Todaro M. p63 role in breast cancer. Aging (Albany NY). 2016; 8:2256–57. 10.18632/aging.101042 27783565 PMC5115884

[23] Gross A, Zaltsman Y, Maryanovich M. The ATM-BID pathway plays a critical role in the DNA damage response by regulating mitochondria metabolism. Cell Death Differ. 2016; 23:182. 10.1038/cdd.2015.154 26611459 PMC4815972

[24] Giacomello M, Pellegrini L. The coming of age of the mitochondria-ER contact: a matter of thickness. Cell Death Differ. 2016; 23:1417–27. 10.1038/cdd.2016.52 27341186 PMC5072433

[25] Kagan VE, Jiang J, Huang Z, Tyurina YY, Desbourdes C, Cottet-Rousselle C, Dar HH, Verma M, Tyurin VA, Kapralov AA, Cheikhi A, Mao G, Stolz D, et al. NDPK-D (NM23-H4)-mediated externalization of cardiolipin enables elimination of depolarized mitochondria by mitophagy. Cell Death Differ. 2016; 23:1140–51. 10.1038/cdd.2015.160 26742431 PMC4946882

[26] Martin-Lopez M, Maeso-Alonso L, Fuertes-Alvarez S, Balboa D, Rodríguez-Cortez V, Weltner J, Diez-Prieto I, Davis A, Wu Y, Otonkoski T, Flores ER, Menéndez P, Marques MM, Marin MC. p73 is required for appropriate BMP-induced mesenchymal-to-epithelial transition during somatic cell reprogramming. Cell Death Dis. 2017; 8:e3034. 10.1038/cddis.2017.432 28880267 PMC5636977

[27] Lovat PE, Ranalli M, Annichiarrico-Petruzzelli M, Bernassola F, Piacentini M, Malcolm AJ, Pearson AD, Melino G, Redfern CP. Effector mechanisms of fenretinide-induced apoptosis in neuroblastoma. Exp Cell Res. 2000; 260:50–60. 10.1006/excr.2000.4988 11010810

[28] Tóth B, Garabuczi E, Sarang Z, Vereb G, Vámosi G, Aeschlimann D, Blaskó B, Bécsi B, Erdõdi F, Lacy-Hulbert A, Zhang A, Falasca L, Birge RB, et al. Transglutaminase 2 is needed for the formation of an efficient phagocyte portal in macrophages engulfing apoptotic cells. J Immunol. 2009; 182:2084–92. 10.4049/jimmunol.0803444 19201861

[29] Zhao J, Yin M, Deng H, Jin FQ, Xu S, Lu Y, Mastrangelo MA, Luo H, Jin ZG. Cardiac Gab1 deletion leads to dilated cardiomyopathy associated with mitochondrial damage and cardiomyocyte apoptosis. Cell Death Differ. 2016; 23:695–706. 10.1038/cdd.2015.143 26517531 PMC4986641

[30] Van Nostrand JL, Bowen ME, Vogel H, Barna M, Attardi LD. The p53 family members have distinct roles during mammalian embryonic development. Cell Death Differ. 2017; 24:575–79. 10.1038/cdd.2016.128 28211873 PMC5384018

[31] Xu-Monette ZY, Zhang S, Li X, Manyam GC, Wang XX, Xia Y, Visco C, Tzankov A, Zhang L, Montes-Moreno S, Dybkaer K, Chiu A, Orazi A, et al. p63 expression confers significantly better survival outcomes in high-risk diffuse large B-cell lymphoma and demonstrates p53-like and p53-independent tumor suppressor function. Aging (Albany NY). 2016; 8:345–65. 10.18632/aging.100898 26878872 PMC4789587

[32] Tak H, Eun JW, Kim J, Park SJ, Kim C, Ji E, Lee H, Kang H, Cho DH, Lee K, Kim W, Nam SW, Lee EK. T-cell-restricted intracellular antigen 1 facilitates mitochondrial fragmentation by enhancing the expression of mitochondrial fission factor. Cell Death Differ. 2017; 24:49–58. 10.1038/cdd.2016.90 27612012 PMC5260506

[33] Dey-Rao R, Sinha AA. Interactome analysis of gene expression profile reveals potential novel key transcriptional regulators of skin pathology in vitiligo. Genes Immun. 2016; 17:30–45. 10.1038/gene.2015.48 26562080

[34] Gianfrancesco MA, Balzer L, Taylor KE, Trupin L, Nititham J, Seldin MF, Singer AW, Criswell LA, Barcellos LF. Genetic risk and longitudinal disease activity in systemic lupus erythematosus using targeted maximum likelihood estimation. Genes Immun. 2016; 17:358–62. 10.1038/gene.2016.33 27467283 PMC5008986

[35] Greschik H, Duteil D, Messaddeq N, Willmann D, Arrigoni L, Sum M, Jung M, Metzger D, Manke T, Günther T, Schüle R. The histone code reader Spin1 controls skeletal muscle development. Cell Death Dis. 2017; 8:e3173. 10.1038/cddis.2017.468 29168801 PMC5775400

[36] Hussman JP, Beecham AH, Schmidt M, Martin ER, McCauley JL, Vance JM, Haines JL, Pericak-Vance MA. GWAS analysis implicates NF-κB-mediated induction of inflammatory T cells in multiple sclerosis. Genes Immun. 2016; 17:305–12. 10.1038/gene.2016.23 27278126 PMC4956564

[37] Salsman J, Rapkin LM, Margam NN, Duncan R, Bazett-Jones DP, Dellaire G. Myogenic differentiation triggers PML nuclear body loss and DAXX relocalization to chromocentres. Cell Death Dis. 2017; 8:e2724. 10.1038/cddis.2017.151 28358373 PMC5386546

[38] Baraibar MA, Hyzewicz J, Rogowska-Wrzesinska A, Bulteau AL, Prip-Buus C, Butler-Browne G, Friguet B. Impaired energy metabolism of senescent muscle satellite cells is associated with oxidative modifications of glycolytic enzymes. Aging (Albany NY). 2016; 8:3375–89. 10.18632/aging.101126 27922824 PMC5270674

[39] Brzeszczyńska J, Johns N, Schilb A, Degen S, Degen M, Langen R, Schols A, Glass DJ, Roubenoff R, Greig CA, Jacobi C, Fearon KC, Ross JA. Loss of oxidative defense and potential blockade of satellite cell maturation in the skeletal muscle of patients with cancer but not in the healthy elderly. Aging (Albany NY). 2016; 8:1690–702. 10.18632/aging.101006 27454226 PMC5032690

[40] Choi JY, Hwang CY, Lee B, Lee SM, Bahn YJ, Lee KP, Kang M, Kim YS, Woo SH, Lim JY, Kim E, Kwon KS. Age-associated repression of type 1 inositol 1, 4, 5-triphosphate receptor impairs muscle regeneration. Aging (Albany NY). 2016; 8:2062–80. 10.18632/aging.101039 27658230 PMC5076452

[41] Fiacco E, Castagnetti F, Bianconi V, Madaro L, De Bardi M, Nazio F, D’Amico A, Bertini E, Cecconi F, Puri PL, Latella L. Autophagy regulates satellite cell ability to regenerate normal and dystrophic muscles. Cell Death Differ. 2016; 23:1839–49. 10.1038/cdd.2016.70 27447110 PMC5071573

[42] Honrath B, Matschke L, Meyer T, Magerhans L, Perocchi F, Ganjam GK, Zischka H, Krasel C, Gerding A, Bakker BM, Bünemann M, Strack S, Decher N, et al. SK2 channels regulate mitochondrial respiration and mitochondrial Ca^2+^ uptake. Cell Death Differ. 2017; 24:761–73. 10.1038/cdd.2017.2 28282037 PMC5423111

[43] Kaufman DM, Wu X, Scott BA, Itani OA, Van Gilst MR, Bruce JE, Crowder CM. Ageing and hypoxia cause protein aggregation in mitochondria. Cell Death Differ. 2017; 24:1730–38. 10.1038/cdd.2017.101 28644434 PMC5596417

[44] Pinto M, Pickrell AM, Wang X, Bacman SR, Yu A, Hida A, Dillon LM, Morton PD, Malek TR, Williams SL, Moraes CT. Transient mitochondrial DNA double strand breaks in mice cause accelerated aging phenotypes in a ROS-dependent but p53/p21-independent manner. Cell Death Differ. 2017; 24:288–99. 10.1038/cdd.2016.123 27911443 PMC5299712

[45] Qi Y, Liu H, Daniels MP, Zhang G, Xu H. Loss of Drosophila i-AAA protease, dYME1L, causes abnormal mitochondria and apoptotic degeneration. Cell Death Differ. 2016; 23:291–302. 10.1038/cdd.2015.94 26160069 PMC4716308

[46] Kaestner L, Minetti G. The potential of erythrocytes as cellular aging models. Cell Death Differ. 2017; 24:1475–77. 10.1038/cdd.2017.100 28622292 PMC5563981

[47] Kanemaru K, Nakamura Y, Totoki K, Fukuyama T, Shoji M, Kaneko H, Shiratori K, Yoneda A, Inoue T, Iwakura Y, Kabashima K, Fukami K. Phospholipase Cδ1 regulates p38 MAPK activity and skin barrier integrity. Cell Death Differ. 2017; 24:1079–90. 10.1038/cdd.2017.56 28430185 PMC5442475

[48] Fortini P, Ferretti C, Iorio E, Cagnin M, Garribba L, Pietraforte D, Falchi M, Pascucci B, Baccarini S, Morani F, Phadngam S, De Luca G, Isidoro C, Dogliotti E. The fine tuning of metabolism, autophagy and differentiation during in vitro myogenesis. Cell Death Dis. 2016; 7:e2168. 10.1038/cddis.2016.50 27031965 PMC4823951

[49] Lee SJ, Hwang J, Jeong HJ, Yoo M, Go GY, Lee JR, Leem YE, Park JW, Seo DW, Kim YK, Hahn MJ, Han JW, Kang JS, Bae GU. PKN2 and Cdo interact to activate AKT and promote myoblast differentiation. Cell Death Dis. 2016; 7:e2431. 10.1038/cddis.2016.296 27763641 PMC5133968

[50] Li G, Luo W, Abdalla BA, Ouyang H, Yu J, Hu F, Nie Q, Zhang X. miRNA-223 upregulated by MYOD inhibits myoblast proliferation by repressing IGF2 and facilitates myoblast differentiation by inhibiting ZEB1. Cell Death Dis. 2017; 8:e3094. 10.1038/cddis.2017.479 28981085 PMC5682648

[51] Palazzo E, Kellett MD, Cataisson C, Bible PW, Bhattacharya S, Sun HW, Gormley AC, Yuspa SH, Morasso MI. A novel DLX3-PKC integrated signaling network drives keratinocyte differentiation. Cell Death Differ. 2017; 24:717–30. 10.1038/cdd.2017.5 28186503 PMC5384032

[52] Shao AW, Sun H, Geng Y, Peng Q, Wang P, Chen J, Xiong T, Cao R, Tang J. Bclaf1 is an important NF-κB signaling transducer and C/EBPβ regulator in DNA damage-induced senescence. Cell Death Differ. 2016; 23:865–75. 10.1038/cdd.2015.150 26794446 PMC4832105

[53] Tajhya RB, Hu X, Tanner MR, Huq R, Kongchan N, Neilson JR, Rodney GG, Horrigan FT, Timchenko LT, Beeton C. Functional KCa1.1 channels are crucial for regulating the proliferation, migration and differentiation of human primary skeletal myoblasts. Cell Death Dis. 2016; 7:e2426. 10.1038/cddis.2016.324 27763639 PMC5133989

[54] Wei X, Li H, Yang J, Hao D, Dong D, Huang Y, Lan X, Plath M, Lei C, Lin F, Bai Y, Chen H. Circular RNA profiling reveals an abundant circLMO7 that regulates myoblasts differentiation and survival by sponging miR-378a-3p. Cell Death Dis. 2017; 8:e3153. 10.1038/cddis.2017.541 29072698 PMC5680912

[55] Zhai L, Wu R, Han W, Zhang Y, Zhu D. miR-127 enhances myogenic cell differentiation by targeting S1PR3. Cell Death Dis. 2017; 8:e2707. 10.1038/cddis.2017.128 28358363 PMC5386531

[56] De Laurenzi V, Melino G. Evolution of functions within the p53/p63/p73 family. Ann N Y Acad Sci. 2000; 926:90–100. 10.1111/j.1749-6632.2000.tb05602.x 11193045

[57] Candi E, Schmidt R, Melino G. The cornified envelope: a model of cell death in the skin. Nat Rev Mol Cell Biol. 2005; 6:328–40. 10.1038/nrm1619 15803139

[58] Candi E, Terrinoni A, Rufini A, Chikh A, Lena AM, Suzuki Y, Sayan BS, Knight RA, Melino G. p63 is upstream of IKK α in epidermal development. J Cell Sci. 2006; 119:4617–22. 10.1242/jcs.03265 17093266

[59] Compagnone M, Gatti V, Presutti D, Ruberti G, Fierro C, Markert EK, Vousden KH, Zhou H, Mauriello A, Anemone L, Bongiorno-Borbone L, Melino G, Peschiaroli A. ΔNp63-mediated regulation of hyaluronic acid metabolism and signaling supports HNSCC tumorigenesis. Proc Natl Acad Sci USA. 2017; 114:13254–59. 10.1073/pnas.1711777114 29162693 PMC5740608

[60] Novelli F, Lena AM, Panatta E, Nasser W, Shalom-Feuerstein R, Candi E, Melino G. Allele-specific silencing of EEC p63 mutant R304W restores p63 transcriptional activity. Cell Death Dis. 2016; 7:e2227. 10.1038/cddis.2016.118 27195674 PMC4917656

[61] Candi E, Amelio I, Agostini M, Melino G. MicroRNAs and p63 in epithelial stemness. Cell Death Differ. 2015; 22:12–21. 10.1038/cdd.2014.113 25168241 PMC4262770

[62] Candi E, Rufini A, Terrinoni A, Dinsdale D, Ranalli M, Paradisi A, De Laurenzi V, Spagnoli LG, Catani MV, Ramadan S, Knight RA, Melino G. Differential roles of p63 isoforms in epidermal development: selective genetic complementation in p63 null mice. Cell Death Differ. 2006; 13:1037–47. 10.1038/sj.cdd.4401926 16601749

[63] Chakrabarti R, Wei Y, Hwang J, Hang X, Andres Blanco M, Choudhury A, Tiede B, Romano RA, DeCoste C, Mercatali L, Ibrahim T, Amadori D, Kannan N, et al. ΔNp63 promotes stem cell activity in mammary gland development and basal-like breast cancer by enhancing Fzd7 expression and Wnt signalling. Nat Cell Biol. 2014; 16:1004–15, 1–13. 10.1038/ncb3040 25241036 PMC4183725

[64] Memmi EM, Sanarico AG, Giacobbe A, Peschiaroli A, Frezza V, Cicalese A, Pisati F, Tosoni D, Zhou H, Tonon G, Antonov A, Melino G, Pelicci PG, Bernassola F. p63 Sustains self-renewal of mammary cancer stem cells through regulation of Sonic Hedgehog signaling. Proc Natl Acad Sci USA. 2015; 112:3499–504. 10.1073/pnas.1500762112 25739959 PMC4372004

[65] Cancer Genome Atlas N, and Cancer Genome Atlas Network. Comprehensive genomic characterization of head and neck squamous cell carcinomas. Nature. 2015; 517:576–82. 10.1038/nature14129 25631445 PMC4311405

[66] Saladi SV, Ross K, Karaayvaz M, Tata PR, Mou H, Rajagopal J, Ramaswamy S, Ellisen LW. ACTL6A Is co-Amplified with p63 in squamous cell carcinoma to drive YAP activation, regenerative proliferation, and poor prognosis. Cancer Cell. 2017; 31:35–49. 10.1016/j.ccell.2016.12.001 28041841 PMC5225026

[67] Campbell JD, Yau C, Bowlby R, Liu Y, Brennan K, Fan H, Taylor AM, Wang C, Walter V, Akbani R, Byers LA, Creighton CJ, Coarfa C, et al, and Cancer Genome Atlas Research Network. Genomic, Pathway Network, and Immunologic Features Distinguishing Squamous Carcinomas. Cell Reports. 2018; 23:194–212.e6. 10.1016/j.celrep.2018.03.063 29617660 PMC6002769

[68] Rocco JW, Leong CO, Kuperwasser N, DeYoung MP, Ellisen LW. p63 mediates survival in squamous cell carcinoma by suppression of p73-dependent apoptosis. Cancer Cell. 2006; 9:45–56. 10.1016/j.ccr.2005.12.013 16413471

[69] Yang X, Lu H, Yan B, Romano RA, Bian Y, Friedman J, Duggal P, Allen C, Chuang R, Ehsanian R, Si H, Sinha S, Van Waes C, Chen Z. ΔNp63 versatilely regulates a Broad NF-κB gene program and promotes squamous epithelial proliferation, migration, and inflammation. Cancer Res. 2011; 71:3688–700. 10.1158/0008-5472.CAN-10-3445 21576089 PMC3443863

[70] Lo Muzio L, Santarelli A, Caltabiano R, Rubini C, Pieramici T, Trevisiol L, Carinci F, Leonardi R, De Lillo A, Lanzafame S, Bufo P, Piattelli A. p63 overexpression associates with poor prognosis in head and neck squamous cell carcinoma. Hum Pathol. 2005; 36:187–94. 10.1016/j.humpath.2004.12.003 15754296

[71] Chen Y, Li Y, Peng Y, Zheng X, Fan S, Yi Y, Zeng P, Chen H, Kang H, Zhang Y, Xiao ZX, Li C. ΔNp63α down-regulates c-Myc modulator MM1 via E3 ligase HERC3 in the regulation of cell senescence. Cell Death Differ. 2018; 25:2118–29. 10.1038/s41418-018-0132-5 29880857 PMC6261956

[72] Si H, Lu H, Yang X, Mattox A, Jang M, Bian Y, Sano E, Viadiu H, Yan B, Yau C, Ng S, Lee SK, Romano RA, et al. TNF-α modulates genome-wide redistribution of ΔNp63α/TAp73 and NF-κB cREL interactive binding on TP53 and AP-1 motifs to promote an oncogenic gene program in squamous cancer. Oncogene. 2016; 35:5781–94. 10.1038/onc.2016.112 27132513 PMC5093089

[73] Carvalho E, Jansson PA, Axelsen M, Eriksson JW, Huang X, Groop L, Rondinone C, Sjöström L, Smith U. Low cellular IRS 1 gene and protein expression predict insulin resistance and NIDDM. FASEB J. 1999; 13:2173–78. 10.1096/fasebj.13.15.2173 10593864

[74] Zhou Y, Zhang Z, Wang N, Chen J, Zhang X, Guo M, John Zhong L, Wang Q. Suppressor of cytokine signalling-2 limits IGF1R-mediated regulation of epithelial-mesenchymal transition in lung adenocarcinoma. Cell Death Dis. 2018; 9:429. 10.1038/s41419-018-0457-5 29559623 PMC5861121

[75] Law JH, Habibi G, Hu K, Masoudi H, Wang MY, Stratford AL, Park E, Gee JM, Finlay P, Jones HE, Nicholson RI, Carboni J, Gottardis M, et al. Phosphorylated insulin-like growth factor-i/insulin receptor is present in all breast cancer subtypes and is related to poor survival. Cancer Res. 2008; 68:10238–46. 10.1158/0008-5472.CAN-08-2755 19074892

[76] Jameson MJ, Beckler AD, Taniguchi LE, Allak A, Vanwagner LB, Lee NG, Thomsen WC, Hubbard MA, Thomas CY. Activation of the insulin-like growth factor-1 receptor induces resistance to epidermal growth factor receptor antagonism in head and neck squamous carcinoma cells. Mol Cancer Ther. 2011; 10:2124–34. 10.1158/1535-7163.MCT-11-0294 21878657 PMC3213311

[77] Chang Q, Li Y, White MF, Fletcher JA, Xiao S. Constitutive activation of insulin receptor substrate 1 is a frequent event in human tumors: therapeutic implications. Cancer Res. 2002; 62:6035–38. 12414625

[78] Koda M, Sulkowska M, Kanczuga-Koda L, Sulkowski S. Expression of insulin receptor substrate 1 in primary breast cancer and lymph node metastases. J Clin Pathol. 2005; 58:645–49. 10.1136/jcp.2004.022590 15917419 PMC1770676

[79] Chan JM, Stampfer MJ, Giovannucci E, Gann PH, Ma J, Wilkinson P, Hennekens CH, Pollak M. Plasma insulin-like growth factor-I and prostate cancer risk: a prospective study. Science. 1998; 279:563–66. 10.1126/science.279.5350.563 9438850

[80] Luo J, Wen Q, Li J, Xu L, Chu S, Wang W, Shi L, Xie G, Huang D, Fan S. Increased expression of IRS-1 is associated with lymph node metastasis in nasopharyngeal carcinoma. Int J Clin Exp Pathol. 2014; 7:6117–24. 25337259 PMC4203230

[81] Dale OT, Aleksic T, Shah KA, Han C, Mehanna H, Rapozo DC, Sheard JD, Goodyear P, Upile NS, Robinson M, Jones TM, Winter S, Macaulay VM. IGF-1R expression is associated with HPV-negative status and adverse survival in head and neck squamous cell cancer. Carcinogenesis. 2015; 36:648–55. 10.1093/carcin/bgv053 25896444

[82] Lara PC, Bordón E, Rey A, Moreno M, Lloret M, Henríquez-Hernández LA. IGF-1R expression predicts clinical outcome in patients with locally advanced oral squamous cell carcinoma. Oral Oncol. 2011; 47:615–19. 10.1016/j.oraloncology.2011.05.005 21640634

[83] Wu X, Zhao H, Do KA, Johnson MM, Dong Q, Hong WK, Spitz MR. Serum levels of insulin growth factor (IGF-I) and IGF-binding protein predict risk of second primary tumors in patients with head and neck cancer. Clin Cancer Res. 2004; 10:3988–95. 10.1158/1078-0432.CCR-03-0762 15217929

[84] Sun JM, Jun HJ, Ko YH, Park YH, Ahn YC, Son YI, Baek JH, Park K, Ahn MJ. Insulin-like growth factor binding protein-3, in association with IGF-1 receptor, can predict prognosis in squamous cell carcinoma of the head and neck. Oral Oncol. 2011; 47:714–19. 10.1016/j.oraloncology.2011.06.007 21708479

[85] Nahor I, Abramovitch S, Engeland K, Werner H. The p53-family members p63 and p73 inhibit insulin-like growth factor-I receptor gene expression in colon cancer cells. Growth Horm IGF Res. 2005; 15:388–96. 10.1016/j.ghir.2005.07.005 16181796

[86] Barbieri CE, Perez CA, Johnson KN, Ely KA, Billheimer D, Pietenpol JA. IGFBP-3 is a direct target of transcriptional regulation by DeltaNp63alpha in squamous epithelium. Cancer Res. 2005; 65:2314–20. 10.1158/0008-5472.CAN-04-3449 15781645

[87] Kouwenhoven EN, van Heeringen SJ, Tena JJ, Oti M, Dutilh BE, Alonso ME, de la Calle-Mustienes E, Smeenk L, Rinne T, Parsaulian L, Bolat E, Jurgelenaite R, Huynen MA, et al. Genome-wide profiling of p63 DNA-binding sites identifies an element that regulates gene expression during limb development in the 7q21 SHFM1 locus. PLoS Genet. 2010; 6:e1001065. 10.1371/journal.pgen.1001065 20808887 PMC2924305

[88] Weinstein JN, Collisson EA, Mills GB, Shaw KR, Ozenberger BA, Ellrott K, Shmulevich I, Sander C, Stuart JM, and Cancer Genome Atlas Research Network. The Cancer Genome Atlas Pan-Cancer analysis project. Nat Genet. 2013; 45:1113–20. 10.1038/ng.2764 24071849 PMC3919969

[89] Candi E, Agostini M, Melino G, Bernassola F. How the TP53 family proteins TP63 and TP73 contribute to tumorigenesis: regulators and effectors. Hum Mutat. 2014; 35:702–14. 10.1002/humu.22523 24488880

[90] Abraham CG, Ludwig MP, Andrysik Z, Pandey A, Joshi M, Galbraith MD, Sullivan KD, Espinosa JM. ΔNp63α Suppresses TGFB2 Expression and RHOA Activity to Drive Cell Proliferation in Squamous Cell Carcinomas. Cell Reports. 2018; 24:3224–36. 10.1016/j.celrep.2018.08.058 30232004 PMC6219633

[91] Tanaka S, Wands JR. Insulin receptor substrate 1 overexpression in human hepatocellular carcinoma cells prevents transforming growth factor beta1-induced apoptosis. Cancer Res. 1996; 56:3391–94. 8758899

[92] Xu H, Lee MS, Tsai PY, Adler AS, Curry NL, Challa S, Freinkman E, Hitchcock DS, Copps KD, White MF, Bronson RT, Marcotrigiano M, Wu Y, et al. Ablation of insulin receptor substrates 1 and 2 suppresses *Kras*-driven lung tumorigenesis. Proc Natl Acad Sci USA. 2018; 115:4228–33. 10.1073/pnas.1718414115 29610318 PMC5910837

[93] Luo J, Feng J, Wen Q, Qoyawayma C, Wang W, Chen L, Lu J, Zhan Y, Xu L, Zang H, Fan S, Chu S. Elevated expression of IRS-1 associates with phosphorylated Akt expression and predicts poor prognosis of breast invasive ductal carcinoma. Hum Pathol. 2018; 79:9–17. 10.1016/j.humpath.2018.03.003 29551677

[94] Matuschek C, Rudoy M, Peiper M, Gerber PA, Hoff NP, Buhren BA, Flehmig B, Budach W, Knoefel WT, Bojar H, Prisack HB, Steinbach G, Shukla V, et al. Do insulin-like growth factor associated proteins qualify as a tumor marker? Results of a prospective study in 163 cancer patients. Eur J Med Res. 2011; 16:451–56. 10.1186/2047-783X-16-10-451 22024424 PMC3400976

[95] Smirnov A, Lena AM, Cappello A, Panatta E, Anemona L, Bischetti S, Annicchiarico-Petruzzelli M, Mauriello M, Melino G, Candi E. ZNF185 is a p63 target gene critical for epidermal differentiation and squamous cell carcinoma development. Oncogene. 2018.Epub ahead of print. 10.1038/s41388-018-0509-4 30337687 PMC6755960

[96] Colaprico A, Silva TC, Olsen C, Garofano L, Cava C, Garolini D, Sabedot TS, Malta TM, Pagnotta SM, Castiglioni I, Ceccarelli M, Bontempi G, Noushmehr H. TCGAbiolinks: an R/Bioconductor package for integrative analysis of TCGA data. Nucleic Acids Res. 2016; 44:e71. 10.1093/nar/gkv1507 26704973 PMC4856967

[97] Lopez-Raton M, Cadarso-Suarez C, Rodriguez-Alvarez MX, Gude-Sampedro F. Optimal cutpoints: an R package for selecting optimal cutpoints in diagnostic tests. J Stat Softw. 2014; 61:1–36. 10.18637/jss.v061.i08

