# Supplementary material for: ΔNp63 promotes IGF1 signalling through IRS1 in squamous cell carcinoma
Source: Aging (Albany NY). 2018 Dec 28;10(12):4224–40. doi: 10.18632/aging.101725 (PMC6326668; doi:10.18632/aging.101725)
Supplement: Supplementary File [file aging-10-101725-s001.pdf]

SUPPLEMENTARY MATERIAL

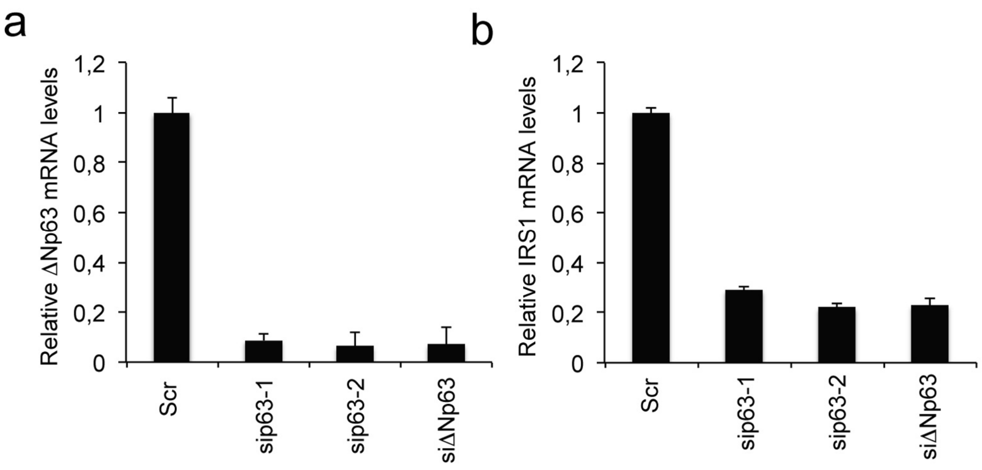

Supplementary Figure 1. Effect of distinct siRNAs against p63 on IRS1 expression.

Supplementary Table S1. Clinical features of HNSCC patients (as provided by Biomax), whose tumour samples were analysed for p63/IRS1 expression levels.

Patient sample	p63 Score	IRS1 Score	Organ	PATHOLOGY DIAGNOSIS	GRADE	STAGE	TNM	TYPE †	SEX	AGE
**A1**	0	0,5	Lip	Squamous cell carcinoma of right lower lip	1	IIA	T2N0M0	Malignant	F	60
**A2**	2	1	Nose	Squamous cell carcinoma of right nasal cavity	1	I	T1N0M0	Malignant	F	45
**A3**	0,5	1	Tongue	Squamous cell carcinoma	1	III	T3N1M0	Malignant	M	28
**A4**	0,5	0,5	Tongue	Squamous cell carcinoma	1	II	T2N0M0	Malignant	M	42
**A5**	1	1	Tongue	Squamous cell carcinoma	1	IIA	T2N0M0	Malignant	M	50
**A6**	1,5	0,5	Larynx	Squamous cell carcinoma	1	II	T2N0M0	Malignant	M	45
**A7**	0,5	1	Larynx	Squamous cell carcinoma	1	IVA	T4N0M0	Malignant	M	49
**A8**	1,5	1	Larynx	Squamous cell carcinoma	1	II	T2N0M0	Malignant	M	66
**A9**	0,5	1,5	Larynx	Squamous cell carcinoma	1	IVA	T4N0M0	Malignant	M	47
**A10**	0,5	0,5	Larynx	Squamous cell carcinoma	–	IV	T4N0M0	Malignant	M	60
**B1**	0,5	0,5	Larynx	Squamous cell carcinoma	1	II	T2N0M0	Malignant	M	51
**B2**	1,5	1,5	Larynx	Squamous cell carcinoma	2	IIB	T2N1M0	Malignant	M	59
**B3**	0,5	2	Cheek	Squamous cell carcinoma of left cheek	1	II	T2N0M0	Malignant	M	37
**B4**	3	3	Face	Squamous cell carcinoma	1	III	T3N0M0	Malignant	M	83
**B5**	1,5	1	Face	Squamous cell carcinoma of mandible	1	III	T3N0M0	Malignant	M	57
**B6**	0	1	Cheek	Squamous cell carcinoma of buccal region	1	II	T2N0M0	Malignant	M	70
**B7**	2	0,5	Oral cavity	Squamous cell carcinoma of gingiva	1	III	T3N0M0	Malignant	M	60
**B8**	0,5	1	Oral cavity	Squamous cell carcinoma of left maxillary sinus	1	III	T3N0M0	Malignant	M	55
**B9**	2,5	0,5	Oral cavity	Squamous cell carcinoma of upper jaw	1	IV	T4N0M0	Malignant	M	40
**B10**	1,5	1	Tongue	Squamous cell carcinoma	1	II	T2N0M0	Malignant	M	58
**C1**	3	0	Larynx	Squamous cell carcinoma	2	IIB	T2N1M0	Malignant	M	45
**C2**	1,5	0,5	Larynx	Squamous cell carcinoma	2	IV	T4N0M0	Malignant	M	50
**C3**	2,5	0	Larynx	Squamous cell carcinoma	2	IV	T2N2M0	Malignant	M	54
**C4**	1	0	Larynx	Squamous cell carcinoma	2	II	T2N0M0	Malignant	M	64
**C5**	3	1	Face	Squamous cell carcinoma of left face	2	I	T1N0M0	Malignant	M	65
**C6**	2,5	0	Larynx	Squamous cell carcinoma	2	IVA	T4N0M0	Malignant	M	49
**C7**	1	0	Larynx	Squamous cell carcinoma	3	I	T1N0M0	Malignant	M	48
**C8**	0	1	Larynx	Squamous cell carcinoma	2	II	T2N0M0	Malignant	M	55
**C9**	2	0,5	Larynx	Squamous cell carcinoma	2	III	T3N0M0	Malignant	M	72
**C10**	1	0	Larynx	Squamous cell carcinoma	3	IV	T4N0M0	Malignant	M	59
**D1**	1	1	Larynx	Squamous cell carcinoma	2	II	T2N0M0	Malignant	M	55
**D2**	1	1	Larynx	Squamous cell carcinoma	2	II	T2N0M0	Malignant	M	54

**D4**	1,5	0,5	Larynx	Squamous cell carcinoma	2	IV	T4N0M0	Malignant	M	65
**D5**	3	0,5	Larynx	Squamous cell carcinoma	2	III	T3N0M0	Malignant	M	75
**D6**	3	0,5	Larynx	Squamous cell carcinoma	2	IVA	T4N1M0	Malignant	M	64
**D7**	3	1,5	Larynx	Squamous cell carcinoma	3	III	T3N1M0	Malignant	F	70
**D8**	1	0	Oral cavity	Squamous cell carcinoma of hypopharynx	3	IV	T1N1M1	Malignant	M	53
**D9**	0	0,5	Larynx	Squamous cell carcinoma	3	III	T2N1M0	Malignant	M	54
**D10**	0	0,5	Nose	Squamous cell carcinoma of sinus piriformis	3	II	T2N0M0	Malignant	M	50
**E1**	1	0,5	Oral cavity	Squamous cell carcinoma of maxillary sinus	3	IIB	T2N1M0	Malignant	F	72
**E2**	1,5	0,5	Oral cavity	Squamous cell carcinoma of left maxillary sinus	3	I	T1N0M0	Malignant	F	51
**E3**	3	1	Larynx	Squamous cell carcinoma	2	IIA	T2N0M0	Malignant	M	71
**E4**	1,5	0	Larynx	Squamous cell carcinoma	3	IIA	T2N0M0	Malignant	F	62
**E5**	3	0,5	Larynx	Squamous cell carcinoma	3	III	T3N0M0	Malignant	M	58
**E6**	2,5	0,5	Larynx	Squamous cell carcinoma of left mandible	3	IIA	T2N0M0	Malignant	M	62
**E7**	2	0,5	Larynx	Squamous cell carcinoma	3	IIA	T2N0M0	Malignant	M	72
**E8**	2	0,5	Larynx	Squamous cell carcinoma	2	IIA	T2N0M0	Malignant	M	73
**E10**	3	0,5	Larynx	Squamous cell carcinoma	3	IVA	T4N0M0	Malignant	M	64
**F1**	3	0	Larynx	Squamous cell carcinoma	2	–	T3N2M0	Malignant	M	57
**F2**	1,5	0,5	Larynx	Squamous cell carcinoma of left nasal cavity	2	III	T3N0M0	Malignant	M	43
**F3**	3	0,5	Larynx	Squamous cell carcinoma	3	III	T3N0M0	Malignant	M	71
**F4**	1,5	0	Larynx	Squamous cell carcinoma	3	IV	T4N1M0	Malignant	M	67
**F5**	3	0	Larynx	Squamous cell carcinoma	3	IV	T3N2M0	Malignant	M	65
**F6**	1,5	0,5	Larynx	Squamous cell carcinoma	3	IV	T4N0M0	Malignant	M	68
**F7**	2	1	Larynx	Squamous cell carcinoma	2	II	T2N0M0	Malignant	M	60
**F8**	2,5	1	Larynx	Squamous cell carcinoma	3	IVA	T4N1M0	Malignant	M	53
**F9**	3	3	Oral cavity	Squamous cell carcinoma of mandible	3	–	–	Malignant	M	56
**F10**	2,5	0	Larynx	Squamous cell carcinoma	3	IV	T4N2M0	Malignant	M	53
**G1**	0	0,5	Larynx	Squamous cell carcinoma of laryngopharynx	3	IV	T4N1M0	Malignant	M	47
**H1**	0,5	0,5	Epiglottis	Epiglottis tissue	–	–	–	Normal	M	28
**H4**	0,5	0,5	Epiglottis	Epiglottis tissue	–	–	–	Normal	F	41
**H4**	3	1	Salivary gland	Salivary gland tissue	–	–	–	Normal	M	22
**H5**	0	0,5	Salivary gland	Salivary gland tissue	–	–	–	Normal	M	22
**H6**	0	0,5	Salivary gland	Salivary gland tissue	–	–	–	Normal	M	43
**H7**	0	0,5	Salivary gland	Salivary gland tissue	–	–	–	Normal	F	15
**H8**	0,5	0,5	Larynx	Larynx tissue	–	–	–	Normal	M	45
**H9**	0,5	0,5	Tongue	Tongue tissue	–	–	–	Normal	M	16
**H10**	0,5	0,5	Tongue	Tongue tissue	–	–	–	Normal	M	48

https://www.biomax.us/tissue-arrays/Head_and_Neck/HN802a
